# Higher‐Order Aberrations, Corneal Density, and Visual Acuity After Excimer Laser Phototherapeutic Keratectomy (PTK) for Epithelial Basement Membrane Dystrophy

**DOI:** 10.1155/joph/4801704

**Published:** 2025-12-30

**Authors:** Simon Helm, Johanna Wiedemann, Benjamin Rosswinkel, Björn Bachmann, Claus Cursiefen, Simona Schlereth

**Affiliations:** ^1^ Department of Ophthalmology, Faculty of Medicine and University Hospital Cologne, Cologne, Germany; ^2^ Institute of Medical Statistics and Computational Biology, Faculty of Medicine and University Hospital Cologne, University of Cologne, Cologne, Germany, uni-koeln.de; ^3^ Department of Ophthalmology and Center for Molecular Medicine Cologne, Faculty of Medicine and University Hospital Cologne, University of Cologne, Cologne, Germany, uni-koeln.de

**Keywords:** corneal haze, epithelial basal membrane dystrophy, HOA, PTK

## Abstract

**Background:**

To evaluate visual acuity outcome after excimer laser phototherapeutic keratectomy (PTK) for epithelial basement membrane dystrophy (EBMD) and to provide new insights into higher‐order aberrations (HOAs) and corneal density changes.

**Methods:**

In this single‐center cohort, 92 eyes from 80 individuals underwent PTK for EBMD, with follow‐up durations reaching up to 7.5 years (mean 332 ± 526.5 days).

**Results:**

Mean BCVA in EBMD improved by 0.17 ± 0.17 logMAR (*p* < 0.001) for patients without visual acuity limitations and by 0.14 ± 0.31 (*p* = 0.004) logMAR if patients had additional visual acuity limitations. Refraction remained stable after PTK. HOA reduced by 0.16 ± 0.23 μm (*p* < 0.001), corneal density by 6.73 ± 11.83 gray scale units (*p* < 0.001), and *K*_max_ by 1.02 dpt (±2.73) (*p* = 0.006) after PTK. A correlation before and after PTK between HOA (*p* = 0.014 and 0.002), corneal density (*p* < 0.002), and *K*_max_ (*p* = 0.010) with BCVA was observed. Light haze occurred in 10% of the cases. Re‐PTK was necessary for 1 patient (1.1%).

**Conclusion:**

PTK significantly enhances visual acuity in patients with EBMD, independently of additional visual acuity limitations. The significant reduction of HOA, corneal density, and *K*_max_ as well as the correlation of these parameters with visual acuity prove an effective therapy on an objective level.

## 1. Introduction

Epithelial basement membrane dystrophy (EBMD) is the most common cause of anterior corneal dystrophy with a prevalence of 2%–6% in the general population [1]. EBMD leads to an uneven thickness of the basement membrane, which in turn causes irregular astigmatism and reduced vision quality [2]. Furthermore, 10% of patients with EBMD develop painful, recurrent corneal erosions. Nevertheless, many patients with EBMD show no symptoms. Due to the characteristic opacities, EBMD is also often found under the alternative name “map‐dot‐fingerprint dystrophy.” In the slit lamp examination, map‐like opacities, epithelial microcysts, and fingerprint‐like lines can be seen, induced by irregular, thickened, and hyperreflective basement membranes, also visible in anterior segment OCT [2]. The majority of cases do not show an inheritance pattern [3, 4].

To treat the symptoms of EBMD and to restore the original shape and clarity of the cornea, excimer laser phototherapeutic keratectomy (PTK) has been a valuable tool since many years [5, 6].

The purpose of this retrospective study was to assess the effectiveness of PTK in treating EBMD for patients with and without additional visual acuity limitations (VALs) and to contribute additional insights into its impact on corneal density and higher‐order aberrations (HOAs) as well as complication and recurrence patterns.

## 2. Methods

The monocentric retrospective cohort study was conducted at the Department of Ophthalmology of the University Hospital Cologne, Germany, and included patients undergoing PTK for visually significant EBMD. The study adhered to the principles of the Declaration of Helsinki. Ethical approval was waived due to the retrospective design.

### 2.1. Inclusion and Exclusion Criteria

Patients with EBMD and treated with PTK due to irregular astigmatism, reduced visual quality, or recurrent corneal erosions, which did not respond to conservative therapy, were included. Eyes with prior corneal refractive surgery, corneal transplantation, or missed follow‐up examination in the University Clinic Cologne were excluded.

### 2.2. Clinical Examinations and Data Collection

All patients received a standardized ophthalmic work‐up prior to surgery. This included slit lamp examination, refraction and BCVA measurement, intraocular pressure assessment, anterior segment OCT, and Scheimpflug tomography (Pentacam HR, Oculus, Germany).

Recorded parameters comprised BCVA (logMAR), subjective refraction, spherical equivalent (SPHQ), corneal power indices (*K*_*m*_, *K*_max_), corneal thickness, HOA, and densitometry. Densitometry values were expressed in grayscale units (GSU), representing increasing corneal light scatter with higher values.

**Figure 1:**
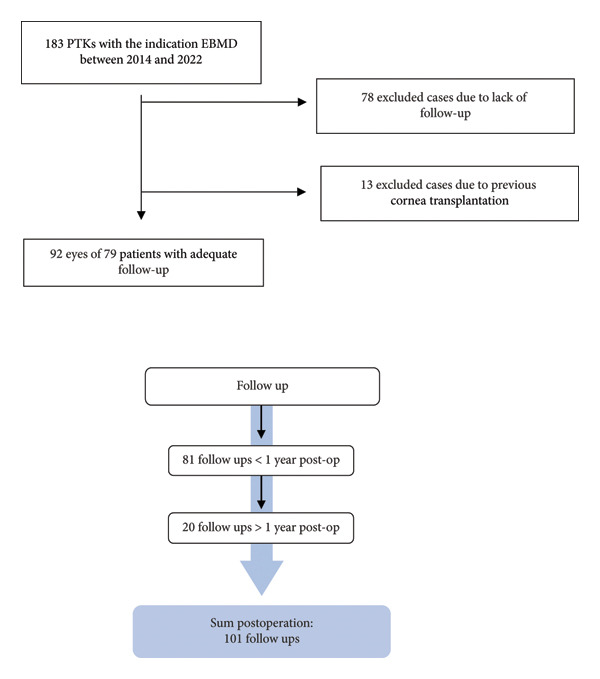
CONSORT‐style flow diagram illustrating the selection of eyes with EBMD treated by PTK. The follow‐up period was categorized into three groups: < 1 year, > 1 year, and total. We recorded 81 follow‐ups within < 1 year and 20 follow‐ups > 1 year postoperatively.

Also, all postoperative complications were documented, including the presence of corneal haze, symptoms of ocular surface dryness, delayed epithelial regeneration, and residuum. A residuum had to be visible in the slit lamp examination and means residual EBMD (residuals after surgery or recurrences). A standardized follow‐up duration was not predefined. Follow‐up visits were categorized according to the interval from surgery to examination into two groups: less than 1 year and more than 1 year postoperatively. For the overall analysis (“total” group), only the latest available follow‐up for each treated eye was included (Figure 1). Eyes were stratified based on the presence of additional VAL considered capable of limiting visual acuity (e.g., cataract or macular disease), as determined by senior clinicians.

Patient information was obtained from the electronic medical record systems Orbis (Dedalus Healthcare, Bonn, Germany) and Fidus (Arztservice Wente, Darmstadt, Germany). All relevant parameters were subsequently organized and processed using Microsoft Excel (Redmond, WA, USA).

### 2.3. Surgical Technique

Initially, the depth of the epithelial lesion was assessed using anterior segment OCT. After topical anesthesia and ocular surface disinfection, patients were placed in a supine position beneath the Schwind Amaris 750S excimer laser system. Mechanical removal of the corneal epithelium together with prominent deposits on Bowman’s layer was performed using a hockey knife over an area of approximately 8 mm in diameter. The laser spot size was 0.7 mm ± 0.05 mm. The mean stromal ablation was 13.9 µm (range: 7–15 µm; see Table 1), and the ablation depth was individualized based on OCT‐derived EBMD thickness measurements. Residual opacities were subsequently treated by excimer laser smoothing using balanced salt solution as a masking agent. Mitomycin‐C 0.02% was then applied topically for 15 s, followed by thorough irrigation. A bandage contact lens was placed and retained until complete epithelial closure. Postoperative care included topical ofloxacin until re‐epithelialization was achieved, as well as dexamethasone eye drops administered four times daily with a gradual taper over four weeks, complemented by lubricating agents. Patients presenting with posterior blepharitis were advised to perform lid hygiene prior to surgery, and those with demodex infestation received appropriate treatment before intervention.

**Table 1:** Descriptive results of eyes with EBMD undergoing PTK showing mean follow‐up time in days (d), mean age, sex category, additional visual acuity limitations, previous eye surgeries, and ablation depth of the excimer laser.

*Mean (±SD) of follow-ups in days (d)*	
< 1 year	115.3 d (±94.4, *n* = 81) min 4 d: max: 336 d
< 1 year	1119.9 (±680.5, *n* = 20) min: 377 d, max: 2867 d
Total	331.6 d (±526.5, *n* = 92) min: 4 d, max: 2867 d
Female	53.3% (*n* = 49)
Male	46.7% (*n* = 43)
Age at intervention (y)	58.4 (±12.7, *n* = 92)

*Visual acuity limitations:*	
None	59.8% (*n* = 55)
One or more causes for additional visual limitation:	40.2% (*n* = 37)
Cataract	28.3% (*n* = 26)
Keratitis sup. punctata and/or dry eye	1.1% (*n* = 1)
Glaucoma	8.7% (*n* = 8)
Retinopathy	7.6% (*n* = 6)
Choroidal atrophy	1.1% (*n* = 1)
Optic neuropathy	2.2% (*n* = 2)
Fuchs endothelia dystrophy	3.3% (*n* = 3)
Amblyopia	4.3% (*n* = 7)
Anisometropia	2.2% (*n* = 2)

*Previous eye surgery due to other eye diseases:*	*13.0% (n = 12)*
Phacoemulsification	13.0% (*n* = 12)
Ablation depth (μm)	13.9 (±4.4, *n* = 76)

### 2.4. Statistical Analysis

All statistical analyses were conducted using IBM SPSS Statistics (Version 29.0.1.0, Chicago, IL, USA). Prior to applying paired *t*‐tests, the differences between pre‐ and postoperative measurements were assessed for normality. A normal distribution was assumed for datasets with sample sizes ≥ 30; for smaller groups (*n* < 30), the Shapiro–Wilk test was performed. In cases where normality was not confirmed, the Wilcoxon signed‐rank test was used as a nonparametric alternative.

One‐sided hypothesis testing was applied for parameters with a predefined expected direction of change. Variables without a clear directional expectation—specifically sphere, cylinder, SPHQ, *K*_*m*_, and *K*_max_—were evaluated using two‐sided tests. Potential associations between continuous parameters were explored using the bivariate correlation analysis with Pearson’s correlation coefficient.

### 2.5. Statistical Test Procedure

All tests were performed with IBM SPSS Statistics (Version 29.0.1.0, Chicago, IL). Before the paired *t*‐test was applied, the difference between the post‐ and pre‐PTK data was checked for normal distribution. Normal distribution was assumed for groups with *n* ≥ 30. For groups *n* < 30, we used the Shapiro–Wilk test to check normal distribution. If the normal distribution was violated, the Wilcoxon nonparametric test procedure was used instead of the paired *t*‐test. For the parameters with an expected direction, we used one‐sided *p*. In the case of sphere, cylinder, SPHQ, *K*_*m*_, and *K*_max_, we used two‐sided *p* due to an unclear direction of change. To analyze a possible correlation, we used the bivariate correlation with the Pearson coefficient.

## 3. Results

This study included 92 eyes of 80 patients with EBMD. The mean age was 58.4 ± 12.7 years. 53.3% (*n* = 49) of the patients were female. Additional visual limitations were present in 40.2% (*n* = 37) of the cases. The most common visual limitation was cataract (28.3%) (Table 1). The mean follow‐up time was 331.6 days ± 526.5 (Figure 1; Table 1).

### 3.1. BCVA (logMAR)

**Figure 2:**
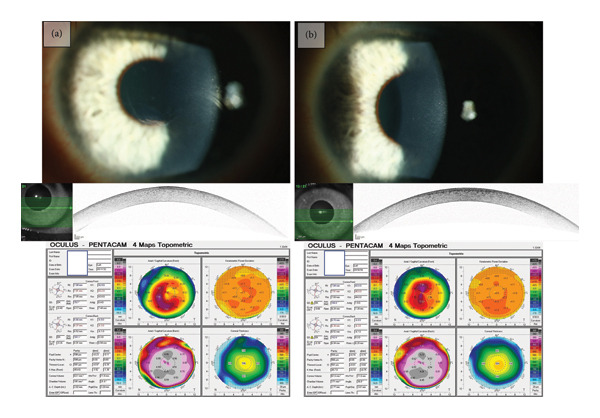
Slit lamp examination, OCT, and Pentacam (Oculus) of a patient with EBMD. (a) Preoperative findings showing fingerprint‐like lines, the opacity in the anterior segment OCT, as well as the irregular astigmatism in the tomography. (b) Slit examination 3 months after PTK. Visual acuity improved from 0.40 logMAR preoperatively to 0.00 logMAR postoperatively, and the anterior segment OCT shows less central irregularities and mild haze, as well as the reduction of irregular astigmatism.

**Figure 3:**
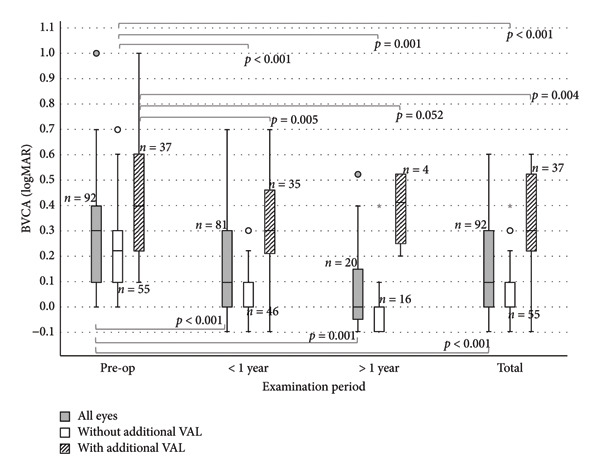
Boxplot diagram showing improvements in visual acuity for eyes with EBMD undergoing PTK: eyes without additional VAL show improvements in all follow‐ups (*p* < 0.001; green boxplot) as well as eyes with additional VAL except in the follow‐up > 1 year (*p* = 0.005, *p* = 0.052, and *p* < 0.004). Both cohorts together (gray boxplot) show significant improvements also in all follow‐ups (*p* < 0.001). Circles (◦) in the colour of the group, are used to identify mild outliers (>1.5 × IQR and ≤3 × IQR), asterisks (∗) mark extreme outliers (>3 × IQR).

Preoperatively, the MV of visual acuity (logMAR) in all eyes was 0.32 ± 0.29 (*n* = 92). At follow‐up < 1 year postoperatively, the MV increased to 0.18 ± 0.23 (*p* < 0.001, *n* = 81). In the follow‐up > 1 year postoperatively, the MV was 0.08 ± 0.20 (*p* < 0.001, *n* = 20). In the entire postoperative follow‐up, the MV was 0.16 ± 0.22 (*p* < 0.001, *n* = 92) (Figures 2 and 3).

Patients without visual limitations started with a visual acuity of preoperatively MV (±SD) 0.21 ± 0.17 logMAR (*n* = 55). At follow‐up < 1 year, the MV of BCVA (logMAR) improved to 0.05 ± 0.09 (*p* < 0.001, *n* = 46). At follow‐up > 1 year, the MV improved to 0.00 ± 0.12 (*p* < 0.001, *n* = 16). In the entire follow‐up, BCVA (logMAR) improved to 0.04 ± 0.10 (*p* < 0.001, *n* = 55).

Patients with additional visual limitations started with an average preoperative visual acuity of (±SD) 0.49 ± 0.34 logMAR (*n* = 37). At follow‐up < 1 year, the MV of BCVA (logMAR) improved to 0.34 ± 0.25 (*p* = 0.005, *n* = 35). At follow‐up > 1 year, the MV improved to 0.39 ± 0.16 (*p* = 0.052, *n* = 4). In the entire follow‐up, BCVA (logMAR) improved to 0.34 ± 0.24 (*p* = 0.004, *n* = 37).

### 3.2. Refraction

**Figure 4:**
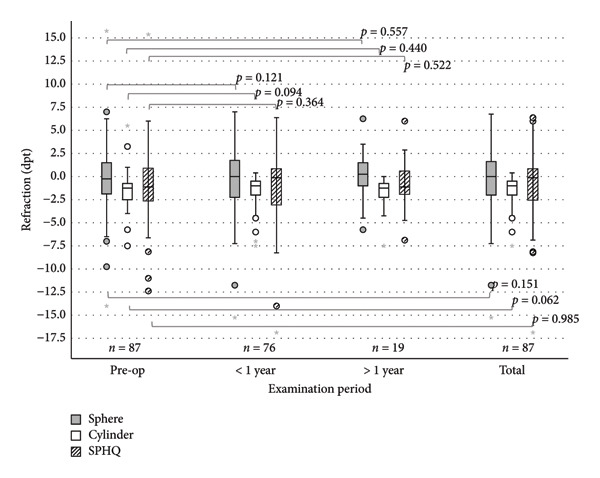
No significant changes in refraction are seen (sphere, cylinder, and SPHQ) in all follow‐ups in eyes with EBMD undergoing PTK. Circles (◦) in the colour of the group, are used to identify mild outliers (>1.5 × IQR and ≤3 × IQR), asterisks (∗) mark extreme outliers (>3 × IQR).

No significant changes were seen in refractive parameters (Figure 4). Preoperatively, the mean (±SD) sphere was −0.04 ± 3.76 dpt (*n* = 87). At follow‐up < 1 year, the MV sphere was −0.39 ± 3.74 dpt (*p* = 0.146, *n* = 76), and in the follow‐up > 1 year, 0.11 ± 2.69 dpt (*p* = 0.557, *n* = 19). In the entire follow‐up, the MV of sphere was −0.36 ± 3.50 dpt (*p* = 0.162, *n* = 87). No hyperopic shift was observed.

The cylinder was preoperatively −1.68 ± 1.49 dpt (*n* = 87). At follow‐up < 1 year, the mean cylinder was −1.43 ± 1.50 dpt (*p* = 0.123, *n* = 76), and in the follow‐up > 1 year, −1.74 ± 1.73 dpt (*p* = 0.297, *n* = 19). In the entire follow‐up, the mean cylinder was −1.40 ± 1.33 dpt (*p* = 0.065, *n* = 87).

The MV of the SPHQ was preoperatively −0.89 ± 3.68 dpt (*n* = 87), and at the follow‐up < 1 year after PTK, ‐1.11 ± 3.86 dpt (*p* = 0.364, *n* = 76). In the follow‐up > 1 year after PTK, the MV of the SPHQ was −0.75 ± 2.76 dpt (*p* = 0.695, *n* = 19). In the total follow‐up, the MW of the SPHQ was −0.90 ± 3.38 dpt (*p* = 0.963, *n* = 87).

### 3.3. *K*_*m*_ and *K*_max_

**Figure 5:**
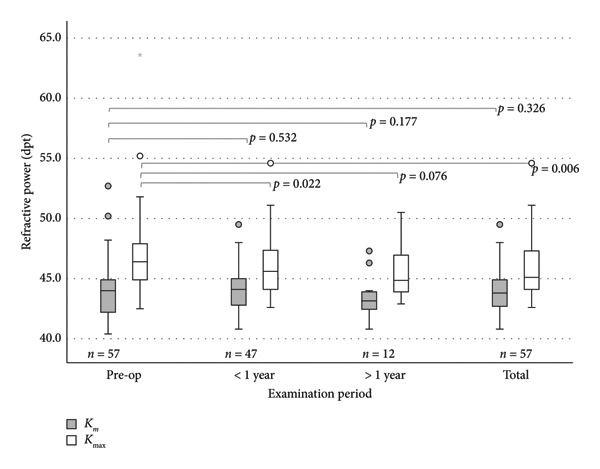
Refractive power in eyes with EBMD undergoing PTK: *K*_max_ shows a significant reduction in the follow‐ups > 1 year and total *K*_*m*_ does not change in a significant way (*p* = 0.532,  *p* = 0.177,  and *p* = 0.326). Circles (◦) in the colour of the group, are used to identify mild outliers (>1.5 × IQR and ≤3 × IQR), asterisks (∗) mark extreme outliers (>3 × IQR).

Preoperatively, the mean (MV) ± SD for *K*_*m*_ was 44.09 ± 2.31 dpt (*n* = 57), and for *K*_max_, 46.79 ± 3.30 dpt (*n* = 57), respectively (Figure 5). The total postoperative MV was 43.92 ± 1.87 dpt (*p* = 0.326, *n* = 57) for *K*_*m*_ and 45.77 ± 2.40 dpt (*p* = 0.006, *n* = 57) for *K*_max_.

While *K*_*m*_ showed no significant differences across all follow‐ups, *K*_max_ decreased significantly. At follow‐up < 1 year, the MV for *K*_*m*_ was 44.06 ± 1.84 dpt (*p* = 0.532, *n* = 47), and at the follow‐up > 1 year, *K*_*m*_ was 43.43 ± 1.85 dpt (*p* = 0.177, *n* = 12). *K*_max_ decreased in the < 1 year follow‐up to 45.85 ± 2.38 dpt (*p* = 0.022, *n* = 47) and stayed stable in the follow‐up > 1 year (mean *K*_max_: 45.58 ± 2.50 dpt [*p* = 0.076, *n* = 12]).

### 3.4. Astigmatism, HOA and Pachymetry

The corneal astigmatism (measured by Scheimflug) did not show significant changes due to the treatment. Preoperatively, the mean (±SD) corneal astigmatism was 1.72 ± 1.17 dpt (*n* = 57); in the follow‐up < 1 year postoperatively, 1.56 ± 1.35 dpt (*p* = 0.090, *n* = 47); and at follow‐up > 1 year, 1.92 ± 1.56 dpt (*p* = 0.246, *n* = 12). The overall postoperative mean corneal astigmatism was 1.57 ± 1.30 dpt (*p* = 0.060, *n* = 57). (Table 2).

**Table 2:** Changes in corneal astigmatism, HOA, and corneal thickness in eyes with EBMD undergoing PTK: HOA shows significant changes in all follow‐ups (*p* < 0.001,  *p* = 0.032,  and *p* < 0.001), whereas no significant change in corneal astigmatism can be seen (*p* = 0.090,  *p* = 0.246,  and *p* = 0.060).

	Preoperatively	< 1 year	> 1 year	Total
Corneal astigmatism (dpt)	1.72 ± 1.17	1.56 ± 1.35	1.92 ± 1.56	1.57 ± 1.30
*n*	57	47	12	57
*p* value		0.090	0.246	0.060
HOA (μm)	0.40 ± 0.25	0.24 ± 0.13	0.26 ± 0.15	0.25 ± 0.13
*n*	52	42	12	52
*p* value		< 0.001	0.032	< 0.001
Corneal thickness at the center (μm)	573.04 ± 50.12	537.55 ± 44.45	553.25 ± 27.47	541.02 ± 42.56
*n*	57	47	12	57
*p* value		< 0.001	< 0.001	< 0.001
Corneal thickness at the thinnest point (μm)	565.37 ± 46.85	534.36 ± 45.10	551.00 ± 26.38	538.02 ± 43.04
*n*	57	47	12	57
*p* value		< 0.001	< 0.001	< 0.001

*Note:* For corneal thickness, a significant reduction is shown at the center as well as at the thinnest point in all follow‐ups (*p* < 0.001).

HOA decreased significantly after PTK (Table 2). Preoperatively, the mean (±SD) HOA was 0.40 μm (±0.25, *n* = 52) and decreased in the follow‐up < 1 year to 0.24 ± 0.13 μm (*p* < 0.001, *n* = 42) and stayed stable in the follow‐up > 1 year (0.26 ± 0.15 μm [*p* = 0.032, *n* = 12]). The overall postoperative mean decreased by almost half to 0.25 ± 0.13 μm (*p* < 0.001, *n* = 52).

The pachymetric values in the corneal center and at the thinnest point of the cornea were compared (Table 2) and as expected significantly reduced: Preoperatively (*n* = 57), the mean corneal thickness (±SD) was 573.04 ± 50.12 μm (*n* = 57) (in the center) and 565.37 ± 46.85 μm (*n* = 57) (at the thinnest point). The mean ablation of the laser was 13.9 μm (±4.4, *n* = 76). At the follow‐up < 1 year, the mean corneal thickness was 537.55 ± 44.45 μm (*p* < 0.001, *n* = 47, center) and 534.36 ± 45.10 μm (*p* < 0.001, *n* = 47, thinnest point). At follow‐up > 1 year, the mean corneal thickness was 553.25 ± 27.47 μm (*p* < 0.001, *n* = 12, center) and 551.00 ± 26.38 μm (*p* < 0.001, *n* = 12, thinnest place). In the total follow‐up, the mean corneal thickness decreased to 541.02 ± 42.56 μm (*p* < 0.001, *n* = 57, center) and to 538.02 ± 43.04 μm (*p* < 0.001, *n* = 57, thinnest place).

### 3.5. Densitometry

**Figure 6:**
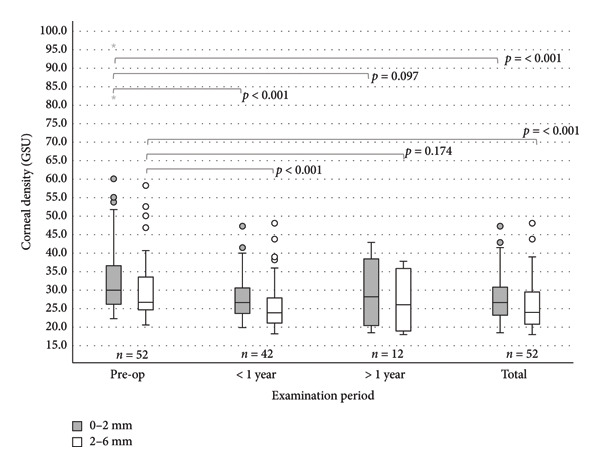
Corneal densitometry in the area 0–2 mm and 2–6 mm in eyes with EBMD undergoing PTK: significant changes in both areas are seen (*p* < 0.001) except in the follow‐up > 1 year (*p* = 0.097 and *p* = 0.174). Circles (◦) in the colour of the group, are used to identify mild outliers (>1.5 × IQR and ≤3 × IQR), asterisks (∗) mark extreme outliers (>3 × IQR).

Corneal densitometry was quantified within two zones of the cornea: the central 0–2 mm and the midperipheral 2–6 mm region. Values are reported in GSUs (Figure 6). Preoperatively, the mean densitometry (±SD) was 34.73 ± 14.05 GSU (*n* = 52) in the center and 30.58 ± 9.29 GSU (*n* = 52) in the midperiphery (compared to published normal values in the center of ∼22 GSU [10] and in the midperiphery of ∼21 GSU [10]). At follow‐up < 1 year, the values decreased significantly to 27.85 ± 5.71 GSU (*p* < 0.001, *n* = 42) and 26.04 ± 6.69 GSU (*p* < 0.001, *n* = 42). At follow‐up > 1 year, the mean was 29.25 ± 9.18 GSU (*p* = 0.097, *n* = 12) in the center and 26.89 ± 8.01 GSU (*p* = 0.174, *n* = 12) in the midperiphery. In the total follow‐up, the mean decreased to 28.00 ± 6.51 GSU (*p* < 0.001, *n* = 52) in the center and 26.09 ± 6.92 GSU (*p* < 0.001, *n* = 52) in the midperiphery.

### 3.6. Correlations

Before and after PTK, there was a significant, linear correlation between the parameters HOA, densitometry, and *K*_max_ with visual acuity (logMAR) for patients with EBMD (Table 3).

**Table 3:** Significant correlations of BCVA (logMAR) with HOA, corneal density, and *K*_max_ are shown for eyes with EBMD prior to and after PTK.

	HOA	Densitometry		*K*_max_
		0–2 mm	2–6 mm	
Before PTK				
Pearson correlation with BCVA (logMAR)	0.372	0.595	0.536	0.380
Significance (1‐sided)	0.014	< 0.001	0.001	0.010
*N*	35	34	34	37
After PTK				
Pearson correlation with BCVA (logMAR)	0.482	0.481	0.381	0.379
Significance (1‐sided)	0.002	0.002	0.013	0.010
*N*	35	34	34	37

### 3.7. Complications

The complication rate was evaluated based on the documented findings at the defined follow‐up visits (Table 4). Descriptors such as “minimal,” “slight,” or “fine” were categorized as mild complications, whereas terms such as “pronounced,” “marked,” or “extensive” were classified as severe. The findings reported without qualifying adjectives were assigned to the moderate category (Table 4). Mild haze was seen in 10% of patients. No moderate or severe haze was seen, probably due to the low ablation depth and intraoperative use of mitomycin C. Keratitis superficial punctate (KSP), as a sign of dry eyes, was seen in 22.5% of the patients, mainly in the first follow‐ups. Severe KSP was seen in 2% of patients, only in the first follow‐ups. Residuum (also only in the periphery) was seen in up to 22% of patients, which further increased in the follow‐ups > 1 year to 31% of patients, indicating the recurrence of the underlying disease. In contrast, re‐PTK was necessary in only 1 patient after 29 months after PTK.

**Table 4:** Complications after PTK for eyes with EBMD: the rate of haze, KSP, dry eye, residuum, wound healing disorders, recurrence, and necessary re‐PTK is shown.

	< 1 year	> 1 year	Total
Haze	10.0% (*n* = 8)	10.5% (*n* = 2)	10.0% (*n* = 9)
Mild	10.0% (*n* = 8)	10.5% (*n* = 2)	10.0% (*n* = 9)
Moderate	0%	0%	0%
Strong	0%	0%	0%
KSP and/or dry eye	22.5% (*n* = 18)	10.5% (*n* = 2)	18.9% (*n* = 17)
Mild	10.0% (*n* = 8)	0%	7.8% (*n* = 7)
Moderate	10.0% (*n* = 8)	10.5% (*n* = 2)	8.9% (*n* = 8)
Strong	2.5% (*n* = 2)	0%	2.2% (*n* = 2)
Residuum	16.3% (*n* = 13)	31.6% (*n* = 6)	21.1% (*n* = 19)
Mild	15.0% (*n* = 12)	26.3% (*n* = 5)	18.9% (*n* = 17)
Moderate	1.3% (*n* = 1)	5.3% (*n* = 1)	2.2% (*n* = 2)
Strong	0%	0%	0%
Wound healing disorder	1.3% (*n* = 1)	0%	1.1% (*n* = 1)
Recurrence	0%	5.3% (*n* = 1)	1.1% (*n* = 1)
Re‐PTK			1.1% (*n* = 1)

*Note:* A residuum had to be visible in the slit lamp examination.

## 4. Discussion

In this study, PTK demonstrated a clear benefit in patients with EBMD, resulting in improved visual acuity and adds new information about the significant reduction in HOA and corneal density in a larger cohort (*n* = 92). Furthermore, this study showed a positive correlation of HOA, corneal densitometry, and *K*_max_ with visual acuity before and after PTK, leading us to conclude that these parameters are objective indicators to show improvement. Both cohorts with and without VAL benefitted in almost the same way from PTK regarding visual acuity.

Independent of additional visual acuity impairment, visual improvement amounts to 0.16 ± 0.23 logMAR (*p* < 0.001) and is similar to the results of a study by Adams et al., in which two laser systems for PTK in EBMD were compared. In that study, the Laser Zeiss MEL 70 performed significantly better than the Schwind Amaris 750S (0.16 vs. 0.09 logMAR, *p* < 0.017) [11]. Similar improvements in visual acuity were also achieved by Lee with an improvement of 0.17 ± 0.19 logMAR [5] (AMO VISX Star, Abbott) and Pogorelov (Zeiss) with 0.11 logMAR [6].

Patients with EBMD presented already close to emmetropia prior to PTK and showed stable refraction (SPHQ) without myopic or hyperopic shift (*p* = 0.963, *n* = 87) after PTK. This is also the consistent result of the studies of Adams, Lee, and Grauvogl [5, 11, 12]. Thus, a hyperopic shift through PTK described in studies by Dogru and Deshmukk cannot be confirmed here [13, 14]. This is most likely due to the low ablation depth and further improvements in the use of modern lasers.

*K*_max_ decreased by an average of 1.02 dpt (±2.73) (*p* = 0.006, *n* = 57) after PTK, showing that preoperative EBMD findings with extremely deviating refractive values could be successfully corrected by PTK. No differences in corneal astigmatism were shown, showing that we do not observe PTK‐induced astigmatism. The missing significant effect on astigmatism has also been described in studies by Adams and Pogorelov [7, 11]. In Adams’ case, there was no significant change in topographic astigmatism in either of the two laser models. Only a significant improvement in the minus cylinder with the Zeiss laser was described from preoperatively 1.58 ± 1.31 dpt to postoperatively 1.04 ± 0.80 dpt. (*p* < 0.042, *n* = 24) [11]. A constant astigmatism after PTK is also postulated in Pogorelov´s study after PTK [6]. The significant reduction in *K*_max_ without any change in *K*_*m*_ or refraction could be due to more localized, stronger steepening caused by the EBMD, which is not reflected in the refraction or *K*_*m*_ across the entire cornea.

HOA were reduced by an average of 0.16 ± 0.23 μm (*p* < 0.001, *n* = 52), representing a normalization of the corneal surface after PTK. To date, no other studies analyzing the effect of PTK on HOA with EBMD are available.

Along with distorted and blurred vision, corneal opacity can be made responsible for reduced visual acuity [9, 10]. To assess corneal opacity in a quantitative way, densitometry can be used as an objective parameter. We observed a significant reduction in corneal density by 6.73 ± 11.83 GSU at the center and 4.49 ± 6.62 GSU at the midperiphery. Although the postoperative values after PTK still differ from the standard values with 28.00 ± 6.51 GSU in the center (normal: ∼22 GSU [10]) and 26.09 ± 6.92 GSU in the midperiphery (normal: ∼21 GSU [10]), PTK can help to normalize corneal density.

In summary, corneal density can be reduced significantly, indicating that corneal vision was partly decreased by higher corneal density and partly by irregular astigmatism. At the time of publication, to the best of our knowledge, there are no other studies that have measured corneal density before and after PTK in EBMD. Significant changes in the corneal thickness in the center and at the thinnest place of the cornea (*p* < 0.001) were seen. After PTK, patients with EBMD showed values that correspond to those of a healthy cornea of 533 ± 53 μm [15]. The average ablation was 13.9 (±4.4, *n* = 76) μm and is comparable to studies by Adams (15–20 μm) and Eschtruth [11, 16]. The actual reduction in corneal thickness after PTK in the entire follow‐up course is 32.02 μm (±22.68) at the center and 27.35 μm (±16.11) at the thinnest point (*p* < 0.001, *n* = 57). Grauvogl et al. came to a similar conclusion in 2022 in which the original ablation depth of 10 μm resulted in a real decrease of 2.3–2.6 times higher after PTK in EBMD [12]. This can possibly be explained by the removal of larger deposits on Bowman’s layer with the hockey knife, which are removed in addition to the laser ablation.

A significant positive correlation between visual acuity and the parameters HOA, densitometry, and *K*_max_ can be observed after PTK. While a direct causal link cannot be established, these results are consistent with the idea that the improvement in visual acuity may be due to the removal of corneal opacity (as indicated by densitometry), the restoration of the cornea’s original, uniform shape (reflected in HOA), and the elimination of areas with extreme refractive power (*K*_max_). In this way, these values provide an objective measure of the therapy’s success.

Mild and reversible haze after PTK was observed in 9 eyes (10.0%). In one case, re‐PTK was performed about 2 years later due to residual scars and irregular astigmatism. 3 patients required an intensifying lubrication therapy with artificial tears due to pronounced dry eye symptoms, including KSP after PTK. The results are largely in line with the observations from the study by Adams et al. in which a recurrence rate of 2.6% is described [11]. In a study by Pogorelov et al., haze was observed in 6 eyes (40%), which was completely reversible in 5 cases [6]. In the study of Lee et al., haze was observed after PTK in 9 of 58 eyes (15.5%) with EBMD. In addition, in this study, there was no significant difference in BCVA in the groups with haze and without haze. Only one patient in the haze group showed a reduced visual acuity of 2 lines. Furthermore, no PTK‐induced ectasia was seen in our study.

Several limitations may have influenced the results of this study. The most relevant factor concerns postoperative follow‐up: A considerable proportion of patients did not return to the University Hospital of Cologne for follow‐up assessments and therefore could not be included. Because many patients face long travel distances, postoperative examinations were frequently performed by local ophthalmologists near their homes. Consequently, only 92 of 180 treated eyes (51%) underwent follow‐up evaluation at our clinic and were eligible for analysis.

Furthermore, a potential selection bias cannot be ruled out, as patients experiencing postoperative symptoms or complications may have been more likely to return to our center. Nevertheless, complications occurred infrequently, and all patients were advised to present for reassessment in case of any new complaints. Another limitation is the variability in follow‐up intervals due to the absence of a predefined schedule.

## 5. Summary

PTK can significantly enhance visual acuity with minimal complications in patients with EBMD. Our study contributes additional data on HOA, *K*_max_, and densitometry, demonstrating a significant reduction of these parameters after PTK with a large cohort. These parameters also correlate positively with visual acuity before and after PTK, confirming the therapy’s effectiveness on an objective level. In addition, the HOA and densitometry parameters can be reliable tools for monitoring changes in EBMD before and after PTK in the clinical setting.

## Conflicts of Interest

The authors declare no conflicts of interest.

## Funding

This research was funded by the DFG SFB 1607/1 2024, Project number 501530074, available online: https://www.crc1607.de (accessed on 1 July 2025); funding acquisition: Björn Bachmann, Claus Cursiefen, and Simona Schlereth and the Center of Molecular Medicine Cologne (CMMC) CAP40 to Simona Schlereth. Open access funding was enabled and organized by Projekt DEAL.

## Data Availability

The data that support the findings of this study are available from the corresponding author upon reasonable request.
